# Extended cost-effectiveness analysis of interventions to improve uptake of diabetes services in South Africa

**DOI:** 10.1093/heapol/czae001

**Published:** 2024-01-22

**Authors:** Heather L Fraser, Isabelle Feldhaus, Ijeoma P Edoka, Alisha N Wade, Ciaran N Kohli-Lynch, Karen Hofman, Stéphane Verguet

**Affiliations:** Health Economics and Health Technology Assessment, School of Health and Wellbeing, University of Glasgow, Clarice Pears Building (Level 3), 90 Byres Road, United Kingdom; SA MRC/Centre for Health Economics and Decision Science—PRICELESS SA, School of Public Health, Faculty of Health Sciences, University of the Witwatersrand, 27 St Andrews Road, Johannesburg 2193, South Africa; Department of Global Health and Population, Harvard T.H. Chan School of Public Health, Boston, MA 02115, USA; Health Economics and Epidemiology Research Office, Department of Internal Medicine, School of Clinical Medicine, Faculty of Health Sciences, University of the Witwatersrand, 32 Princess of Wales Terrace, Johannesburg 2193, South Africa; School of Public Health, Faculty of Health Sciences, University of the Witwatersrand, 27 St Andrews Road, Johannesburg 2193, South Africa; MRC/Wits Rural Public Health and Health Transitions Research Unit, School of Public Health, Faculty of Health Sciences, University of the Witwatersrand, 27 St Andrews Road, Johannesburg 2193, South Africa; Division of Endocrinology, Diabetes and Metabolism, Perelman School of Medicine, University of Pennsylvania, 3400 Civic Center Boulevard, Philadelphia, PA 19104, United States; SA MRC/Centre for Health Economics and Decision Science—PRICELESS SA, School of Public Health, Faculty of Health Sciences, University of the Witwatersrand, 27 St Andrews Road, Johannesburg 2193, South Africa; Department of Preventive Medicine, Feinberg School of Medicine, Northwestern University, 680 N. Lake Shore Drive, Chicago, IL 60611, United States; SA MRC/Centre for Health Economics and Decision Science—PRICELESS SA, School of Public Health, Faculty of Health Sciences, University of the Witwatersrand, 27 St Andrews Road, Johannesburg 2193, South Africa; Department of Global Health and Population, Harvard T.H. Chan School of Public Health, Boston, MA 02115, USA

**Keywords:** Economic evaluation, equity, cost-effectiveness analysis, non-communicable disease

## Abstract

The rising prevalence of diabetes in South Africa (SA), coupled with significant levels of unmet need for diagnosis and treatment, results in high rates of diabetes-associated complications. Income status is a determinant of utilization of diagnosis and treatment services, with transport costs and loss of wages being key barriers to care. A conditional cash transfer (CCT) programme, targeted to compensate for such costs, may improve service utilization. We applied extended cost-effectiveness analysis (ECEA) methods and used a Markov model to compare the costs, health benefits and financial risk protection (FRP) attributes of a CCT programme. A population was simulated, drawing from SA-specific data, which transitioned yearly through various health states, based on specific probabilities obtained from local data, over a 45-year time horizon. Costs and disability-adjusted life years (DALYs) were applied to each health state. Three CCT programme strategies were simulated and compared to a ‘no programme’ scenario: (1) covering diagnosis services only; (2) covering treatment services only; (3) covering both diagnosis and treatment services. Cost-effectiveness was reported as incremental net monetary benefit (INMB) using a cost-effectiveness threshold of USD3015 per DALY for SA, while FRP outcomes were reported as catastrophic health expenditure (CHE) cases averted. Distributions of the outcomes were reported by income quintile and sex. Covering both diagnosis and treatment services for the bottom two quintiles resulted in the greatest INMB (USD22 per person) and the greatest CHE cases averted. There were greater FRP benefits for women compared to men. A CCT programme covering diabetes diagnosis and treatment services was found to be cost-effective, when provided to the poorest 40% of the SA population. ECEA provides a useful platform for including equity considerations to inform priority setting and implementation policies in SA.

Key messages

- Extended cost-effectiveness analyses are useful in determining the distributional benefits of healthcare programmes, as well as the financial risk protection benefits.
- A conditional cash transfer programme that covers diagnostics and treatment services was found to be cost-effective in South Africa when provided to the poorest 40% of the population.
- The conditional cash transfer programme was more cost-effective in women compared to men and provides more financial risk protection benefits to women, compared to men.

## Introduction

The global trend in increasing diabetes prevalence disproportionately affects low- and middle-income countries (LMICs), and South Africa is no exception (Zhou *et al*., 2016; Erzse *et al*., 2019). While there is a paucity of recent surveillance data in the country, it has been estimated that diabetes prevalence was approximately 22% in 2016, a notable increase from the 6% estimated in 2000 (Bradshaw *et al*., 2007; Grundlingh *et al*., 2022).

This escalating burden is already placing a strain on the country’s health system and economy, with previous research estimating that the South African health system could be facing an economic burden of approximately $2.5 billion resulting from type-2 diabetes mellitus (T2DM) and associated complications by 2030 (Erzse *et al*., 2019). There is a significant level of unmet need for management of diabetes in South Africa throughout the care continuum, from screening to the retention of patients on diabetes medication. Approximately 41% of people with diabetes are diagnosed, with only 19% of people with diabetes estimated to achieve glycaemic control (Stokes *et al*., 2017). A recent cross-sectional study of the diabetes care cascade in sub-Saharan Africa revealed similar findings: in participants recruited from South African sites, glycaemic control rates were found to be between 19% and 27% for those with diabetes (Wade *et al*., 2023). This low rate of glycaemic control results in higher levels of diabetes-related complications, including retinopathy, nephropathy, peripheral vascular disease, cardiovascular and cerebrovascular disease and death (American Diabetes Association, 2012; Zhou *et al*., 2016; Stokes *et al*., 2017; Erzse *et al*., 2019).

A large proportion of the burden of managing diabetes-related complications falls on the public healthcare sector in South Africa. The majority of the South African population (approximately 84%) receives healthcare services through the public system, for which the cost is heavily subsidized, while a small proportion of the population accesses the private sector, largely through medical insurance (National Department of Health, 2017; Statistics South Africa, 2018). While healthcare is free of charge at primary healthcare facilities, and there are income-related exemptions at public hospitals, patients requiring hospitalization for diabetes-related complications do face out-of-pocket (OOP) direct medical costs if they are unable to obtain exemptions (National Department of Health, 2002; Goudge *et al*., 2009; Mutyambizi *et al*., 2019b). Full exemptions for direct medical costs are available for recipients of social grants and those formally unemployed; while employed individuals earning less than the 80th percentile pay 20% of consultation fees and 1% of inpatient ward tariffs set by the Uniform Patient Fee Schedule (National Department of Health, 2002).

Some of the barriers to accessing diabetes treatment services in South Africa may not apply to diagnosis services. For instance, medication stock-outs at clinics were reported as a barrier to accessing diabetes treatment from public facilities in the Western Cape, and this would not be a barrier to diagnosis (Mendenhall and Norris, 2015). Likewise, barriers to screening and diagnosis, such as lack of knowledge about diabetes or care-seeking behaviour, may not be as large a factor in accessing treatment once diagnosed. However, financial barriers to accessing care are likely to be a barrier to attending services for diagnosis as well as treatment for diabetes, as they are both delivered at primary healthcare facilities, which require transport and time off work to access (Macha *et al*., 2012; Mutyambizi *et al*., 2019b). Research shows that income status is a determinant for access to South African healthcare services, with transport costs and loss of wages due to illness emerging as key barriers (Goudge *et al*., 2009; Harries *et al*., 2010; Harris *et al*., 2011; Macha *et al*., 2012; McLaren *et al*., 2013; Abrahams *et al*., 2019). Local studies have found that transport costs contribute 40–50% of OOP costs for hospitalized patients, and 78% of OOP cost for outpatients utilizing public healthcare services, while 22% of OOP costs for outpatients are attributed to food costs (Goudge *et al*., 2009; Mutyambizi *et al*., 2019b). Recommendations from these studies include the implementation of targeted interventions to reduce cost of transport for patients seeking diabetes care, in order to improve uptake of these services (Mutyambizi *et al*., 2019b).

A conditional cash transfer (CCT) programme could be considered such an intervention, targeted to alleviate the direct non-medical costs and indirect costs faced by patients when attending diabetes-related healthcare services (Mutyambizi *et al*., 2019b; Basu *et al*., 2021). CCTs have been shown to increase healthcare utilization in a number of LMIC settings, through reducing the financial barrier to healthcare access (Lagarde *et al*., 2007; Owusu-Addo *et al*., 2018). These CCT programmes in Africa have been predominantly centred on increasing utilization of immunization, antenatal and HIV services; however, it may be an appropriate intervention to consider for improving uptake of diabetes diagnosis and retention in care, particularly for those in lower-income quintiles (Owusu-Addo *et al*., 2018). A review by the World Bank of cash transfers in sub-Saharan Africa found that there is a growing body of programmes across the continent, with new technology improving feasibility by reducing capacity constraints and identifying eligible individuals. South Africa’s legislative framework for social protection, as well as its existing array of unconditional grants, provide an indication of feasibility for CCT programmes (Garcia *et al*., 2012). Unconditional cash transfer programmes in South Africa include the Child Support Grant, one of the largest unconditional cash transfer programmes in Africa (Plagerson *et al*., 2011; Luthuli *et al*., 2022). While introducing conditionality into cash transfer programmes may prove administratively complex, a randomized controlled trial investigating a CCT programme for school attendance indicates that a CCT programme may be feasible in South Africa (Pettifor *et al*., 2005).

One key objective of the South African National Development Plan is to improve equity in access to healthcare (South Africa National Planning Commission, 2011). Therefore, to assess the impact of new healthcare interventions on equity goals, an understanding of how benefits of the new interventions are distributed across sub-populations, defined along important equity lines such as income and sex, is crucial for informing policy decisions. While traditional cost-effectiveness analyses are useful for assessing the value-for-money of health interventions, they are limited in assessing the equity considerations of health interventions. Extended cost-effectiveness analysis (ECEA) goes one step further, in investigating the distribution of the benefits of treatments among subgroups (e.g. per socioeconomic status) and the financial risk protection (FRP) benefits that an intervention is able to provide (Verguet *et al*., 2016).

The aim of this study was to pursue an ECEA of three different CCT programme strategies to increase uptake of diabetes services in South Africa, compared to a ‘no programme’ scenario. The three programme strategies comprise of CCTs covering (1) diagnosis services only, (2) treatment services only or (3) both diagnosis and treatment services. The objectives were to investigate the distribution of the costs and benefits of a CCT programme among South Africa’s two lowest income quintiles; to estimate the FRP attributes of such an intervention; to estimate the total cost of the intervention; to estimate the total health gains yielded by the intervention.

This study focuses on the lowest two income quintiles, based on research findings which suggest that the odds of diabetes-related impoverishment were highest for the first two income quintiles (Mutyambizi *et al*., 2019b). These impoverishments were caused by direct non-medical costs (largely transport costs); indirect costs from the loss of wages associated with seeking care and time taken off work due to diabetes and related complications; and direct medical costs for patients requiring hospitalization for diabetes-related complications and who were unable to obtain an exemption in the public sector (Mutyambizi *et al*., 2019b).

## Methods

Updated guidelines for analysis and reporting of economic evaluation were followed, with a completed CHEERS checklist available in the Supplementary Appendix (Husereau *et al*., 2022).

### Intervention structure

A CCT programme was envisaged to work alongside the South African Social Security Agency (SASSA) system to provide cash transfers on attendance at diabetes services, designed to offset the cost of transport and lost wages associated with attending healthcare services.

SASSA is responsible for managing and administrating social grants in the country, including the Child Support Grant and Disability Grant. These grants are means-tested, which indicates that SASSA has the system capacity to determine eligibility for a social programme based on income, such as the CCT programme proposed in this study. While a structure does not yet exist for healthcare facilities to administer grants or social programmes, it is envisaged that upon utilizing diabetes diagnosis or treatment services at a healthcare facility, a SASSA credit can be generated using the patient’s identity document and paid directly into their bank account. Undiagnosed patients would be eligible to attend diabetes screening services once per year, and if diagnosed with diabetes, would be eligible to attend all scheduled clinic appointments in line with existing South African clinical guidelines (National Department of Health, 2018a). All residents of South Africa are eligible to receive these services free of charge at primary healthcare facilities, and the CCT programme is intended to support these existing services by alleviating financial barriers to these services for those in the two lowest income quintiles (National Department of Health, 2002).

The temporary Social Relief Distress (SRD) grant, originating during the response to the COVID-19 pandemic, is a form of basic income support provided to South African residents receiving no other income (South African Government, 2020). The mechanism to validate and approve SRD applications—with submission of identity number, bank details and other supporting documents via WhatsApp—could be utilized for the CCT programme, with the addition of a validation note from the healthcare facility of the conditions of the cash transfer being met. This system would also provide the mechanism for direct payment into bank accounts.

Three CCT programme strategies were modelled, based on the services for which patients could receive a CCT. In the first strategy, a CCT would be provided to those attending screening and diagnosis services only; in the second, a CCT would be provided to those attending diabetes treatment services (to receive drug therapy); in the third, a CCT would be provided to those attending both diagnosis and treatment services. Two eligibility scenarios were modelled, the first, where only the lowest income quintile was eligible, and the second, where the lowest two income quintiles were eligible for the CCT programme. The costs and health benefits of the three different CCT programme strategies were compared to a ‘no programme’ scenario. It is important to note that the ‘no programme’ scenario incorporates the current strategies for diagnosis and treatment of diabetes in South Africa, thus while no CCT programme is modelled, individuals in this scenario would utilize diagnosis and treatment services according to current utilization rates.

### Model structure

**Figure 1.**
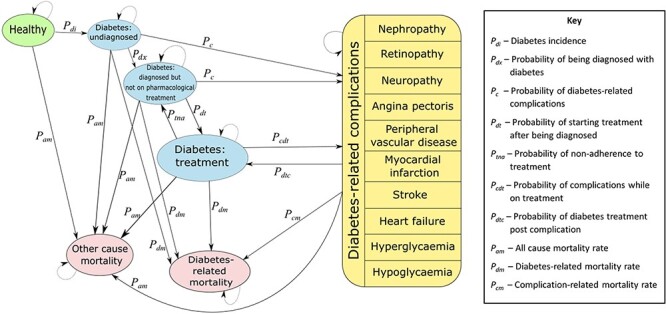
Simplified representation of the adapted Markov model. Adapted from: Feldhaus *et al*., (2021) ‘Alleviating the burden of diabetes with Health Equity Funds: Economic evaluation of the health and financial risk protection benefits in Cambodia’, PLOS ONE, 16(11), p. e0259628. Licenced under Creative Commons Attribution (CC BY 4.0) available at: https://reativecommons.org/licenses/by/4.0/

A Markov model (Figure 1) previously developed by Feldhaus *et al.* to conduct an ECEA for diabetes interventions in Cambodia was updated to model a CCT programme designed to increase utilization of diabetes services in South Africa for the lowest two income quintiles (Feldhaus *et al*., 2021). A population of 12 million individuals was simulated, representing the South African population aged between 25 to 69 years in 2020 in the two lowest income quintiles (Knoema, 2020; Statistics South Africa, 2020b). A microsimulation was run on this population, and cost and health consequences of the CCT programme strategies were calculated from a health system perspective, compared to a ‘no programme’ scenario. FRP benefits were calculated including OOP expenditures and loss of wages (Sanders *et al*., 2016). The model was run over a 45-year time horizon, with cycles of one year, consistent with the original model developed (Feldhaus *et al*., 2021).

A Markov chain was constructed based on an individual’s characteristics and transition probabilities between states, consistent with Feldhaus *et al.* (Feldhaus *et al*., 2021). Following consultation with local clinical experts, two diabetes-related complication health states were added to the original model: severe hypoglycaemia and severe hyperglycaemia. Aside from these additions, the structure of the transition matrix was unaltered from the parent model (Figure 1). The initial state for individuals in the model was determined by South African population characteristics including age, sex and income. Transition to other states in subsequent cycles is determined by the transition matrix and associated probabilities (Feldhaus *et al*., 2021).

When the CCT programme covers diagnosis services, it increases the probability of an individual with undiagnosed diabetes being diagnosed (*P_dx_* in the model), which also improves the likelihood of that person receiving treatment (*P_dt_* in the model), but does not impact the rate of treatment adherence. When the CCT programme covers treatment services, it increases the rate of treatment adherence, improving the likelihood that an individual remains in the ‘diabetes: treatment’ health state (reducing *P_tna_* in the model). As an individual in the treatment state has a lower risk of diabetes-related complications and mortality compared to those not on pharmacological treatment, improving the probability of individuals moving into (and remaining in) the diabetes treatment state is hypothesized to avert disability-adjusted life years (DALYs) associated with diabetes-related complications and mortality. Furthermore, averting diabetes-related complications would reduce the associated direct medical and OOP costs, likely off-setting some of the costs associated with the CCT programme and resulting in FRP benefits.

### Model inputs

The parameters used in the Markov model are described below, with further details in the accompanying tables and the Supplementary Appendix Tables S1–S4. Baseline characteristics of the population are presented in Table 1. These values were derived from the South African Demographic and Health Survey and SANHANES-1 (Shisana *et al*., 2014; National Department of Health, Statistics South Africa (Stats SA), South African Medical Research Council (SAMRC), and ICF, 2019) and helped to determine an individual’s initial state within the model.

**Table 1.** Model Parameters

Health state	Incidence	Effect of diabetes therapy	Direct Medical Cost† (USD 2020)	OOP Cost (USD 2020)†	Disability weight	Sources††
Healthy	n/a	n/a	n/a	n/a	0	
Diabetes:	Age-, sex-and income-specific					(Institute for Health Metrics and Evaluation, 2015)
Undiagnosed	0.600	n/a	n/a	n/a	0.049	(Institute for Health Metrics and Evaluation, 2017; Stokes *et al*., 2017)
Diagnosed and not on pharmacological treatment	0.007	n/a	23	13	0.049	(Institute for Health Metrics and Evaluation, 2017; Stokes *et al*., 2017; National Department of Health, 2018b; National Health Laboratory Service, 2018; Diabetes South Africa, 2019; Tempia *et al*., 2019; National Department of Health South Africa, 2020)
Diagnosed and on oral therapy	0.196	n/a	185	51	0.049	(Institute for Health Metrics and Evaluation, 2015; Pinchevsky *et al*., 2017; Stokes *et al*., 2017; National Department of Health, 2018a, National Department of Health, 2018b, National Department of Health, 2020; Basu *et al*., 2019; Tempia *et al*., 2019)
Diagnosed and on insulin therapy	0.036	n/a	277	51	0.049	(National Department of Health, 2014, National Department of Health, 2018a, National Department of Health, 2020; Institute for Health Metrics and Evaluation, 2017; Pinchevsky *et al*., 2017; Stokes *et al*., 2017; Tempia *et al*., 2019)
Diagnosed and on combination therapy^§^	0.160	n/a	301	51	0.049	(National Department of Health, 2014, National Department of Health, 2018a, National Department of Health, 2020; Institute for Health Metrics and Evaluation, 2017; Pinchevsky *et al*., 2017; Stokes *et al*., 2017; Basu *et al*., 2019; Tempia *et al*., 2019; National Department of Health South Africa, 2020)
Complications:						
Nephropathy	0.010*	0.30	1207	90	1**	(Erzse *et al*., 2019; Mutyambizi *et al*., 2019b; Tempia *et al*., 2019; Feldhaus *et al*., 2021)
Retinopathy	0.021*	0.68	56	13	0.184	(Institute for Health Metrics and Evaluation, 2017; Basu *et al*., 2019; Tempia *et al*., 2019; Feldhaus *et al*., 2021)
Neuropathy	0.047*	0.94	6340	13	0.133	(Institute for Health Metrics and Evaluation, 2017; Basu *et al*., 2019; Tempia *et al*., 2019; Feldhaus *et al*., 2021)
Angina pectoris	0.0067*	0.68	16	13	0.080	(Institute for Health Metrics and Evaluation, 2017; Tempia *et al*., 2019; National Department of Health, 2020; National Department of Health South Africa, 2020; Feldhaus *et al*., 2021)
Peripheral vascular disease	0.0085*	0.74	16	13	0.014	(Institute for Health Metrics and Evaluation, 2017; Tempia *et al*., 2019; National Department of Health, 2020; National Department of Health South Africa, 2020; Feldhaus *et al*., 2021)
Myocardial infarction	0.017*	0.61	1291	90	0.432	(Institute for Health Metrics and Evaluation, 2017; Basu *et al*., 2019; Mutyambizi *et al*., 2019b; Tempia *et al*., 2019; Feldhaus *et al*., 2021)
Stroke	0.0053*	0.59	2168	90	0.588	(Institute for Health Metrics and Evaluation, 2017; Basu *et al*., 2019; Mutyambizi *et al*., 2019b; Tempia *et al*., 2019; Feldhaus *et al*., 2021)
Heart failure	0.0033*	0.68	2493	90	0.179	(Institute for Health Metrics and Evaluation, 2017; Basu *et al*., 2019; Mutyambizi *et al*., 2019b; Tempia *et al*., 2019; Feldhaus *et al*., 2021)
Hyperglycaemia	0.18*	1	83	90	0.133	(Umpierrez and Korytkowski, 2016; Institute for Health Metrics and Evaluation, 2017; Mutyambizi *et al*., 2019b; Tempia *et al*., 2019; National Department of Health, 2020; National Department of Health South Africa, 2020)
Hypoglycaemia	0.18	0.68	76	90	0.133	(UKPDS 34, 1998; Umpierrez and Korytkowski, 2016; Institute for Health Metrics and Evaluation, 2017; Mutyambizi *et al*., 2019b; Tempia *et al*., 2019; National Department of Health, 2020; National Department of Health South Africa, 2020)
Diabetes-related mortality	Age-, sex-and income-specific	n/a	n/a	n/a	1	(Institute for Health Metrics and Evaluation, 2015)
Other-cause mortality	Age- and sex-specific	n/a	n/a	n/a	1	(World Health Organization, 2019)

† Annual costs per person.

†† Sources listed in order of their use through the columns.

§ Combination therapy refers to use of both oral and insulin therapy.

* Incidence of complication for patient with untreated diabetes.

** Due to dialysis only being available for reversible kidney failure (expert advice), patients with diabetes-related nephropathy are not eligible for treatment in the public sector.

All-cause mortality for South Africa, in five-year age groups, was derived from the WHO Global Health Observatory, while age-, sex- and income-specific diabetes incidence and mortality rates for South Africa were derived from the 2017 Global Burden of Disease Study and SANHANES-1 (Shisana *et al*., 2014; Institute for Health Metrics and Evaluation, 2017; World Health Organization, 2019). These rates determined the transition probabilities for an individual moving between the health states in the model.

An individual’s income was simulated based on a gamma distribution informed by the income quintile ranges for South Africa (Southern Africa Labour and Development Research Unit, 2016; Saxena *et al*., 2019), and this determined the individual’s financial risk resulting from diabetes-related illness, in terms of impoverishment or catastrophic health expenditure (CHE), and it is further explained below. South African-specific rates were used to determine the probability of healthcare utilization, and the probability of receiving a specific diabetes therapy (Shisana *et al*., 2014; Pinchevsky *et al*., 2017; Stokes *et al*., 2017; Erzse *et al*., 2019). Income parameters, including income quintile ranges can be found in Supplementary Appendix Table S4.

### Intervention effect

A literature search was conducted to estimate the effect size of the CCT programme on healthcare utilization and treatment adherence rates. In absence of South African-specific evaluations of comparable CCT programmes, a systematic review that synthesized the evidence of CCT interventions in sub-Saharan Africa was used. This study found large variation in the effect size of CCT interventions on healthcare utilization (Owusu-Addo *et al*., 2018). Furthermore, there were differences in the target populations of the studies included in the review. Many evaluated CCT programmes targeted to improve child healthcare utilization. There are differences in the care-seeking behaviour of mothers concerning their children, compared to the care-seeking behaviour of an independent adult. This would likely lead to variation in the effectiveness of CCT programmes for the different target populations.

In order to optimize the generalizability of the intervention effect to our study context, the following three criteria were used to determine inclusion of a study’s estimate: (1) probability of healthcare utilization used as the study outcome; (2) a target population consisting of adults; (3) specification of a CCT programme, rather than unconditional cash transfers. This resulted in only three estimates of intervention effect being included, for which a median odds ratio of 1.31 (95% confidence interval (CI): 1.12–1.54) was taken. These studies evaluated CCT programmes aiming to increase utilization of prevention of mother-to-child HIV transmission (PMTCT) services and antenatal services (Owusu-Addo *et al*., 2018). There were no studies focused on chronic or non-communicable diseases, which may limit generalizability of these treatment effects to the current study. In the absence of differential estimates of CCT programme effectiveness for diagnosis compared to treatment services from the literature, the same intervention effect was assumed across the CCT programme strategies modelled. This was based on both diagnosis and treatment services being delivered in primary healthcare facilities, and the financial barriers to accessing care at primary healthcare facilities, which the CCT was designed to address (i.e. transport costs and loss of wages), being the same whether visiting the facility for diagnosis or treatment services.

### Costs

Costs were attributed to each health state, according to the cost ingredients included in the original model (Feldhaus *et al*., 2021). The costs used in the model are displayed in Table 1, separated into direct medical costs and individual OOP costs, with further details given in Supplementary Appendix Table S1. Costs of screening, diagnosis and treatment were obtained from a study on the direct medical cost of diabetes in South Africa, along with expert clinical advice, the Uniform Patient Fee Schedule, the National Health Laboratory Service catalogue and the Standard Treatment Guidelines (National Health Laboratory Service, 2018; National Department of Health, 2018a; Erzse *et al*., 2019; National Department of Health South Africa, 2020). Cost of diagnosis includes screening (a fasting plasma glucose test), a glycated haemoglobin test (HbA1c) and the facility fee for an outpatient visit at a primary healthcare facility.

Cost of treatment includes the cost of either oral anti-diabetic medication, insulin or both (combination therapy), along with the annual costs associated with ongoing management of patients with diabetes. This includes an outpatient visit every three months; haemoglobin level checks every three months; lipid laboratory tests; annual electrolyte and urea laboratory tests; statins, aspirin, ACE inhibitors and retinopathy screening. Costs associated with each diabetes-related complication were based on the cost ingredients identified by Feldhaus *et al*., 2021 and adapted using South African-specific literature, the Uniform Patient Fee Schedule, Master Procurement Catalogue, as well as clinical expert advice (National Department of Health, 2018b; 2020; Basu *et al*., 2019; Erzse *et al*., 2019).

Individual OOP costs include the direct non-medical costs associated with seeking treatment (such as transport) as well as indirect costs such as loss of wages. In the absence of wage loss estimates specific to accessing diabetes services, estimates of wage loss associated with outpatient care and hospitalization from South African literature were used (Tempia *et al*., 2019; Mutyambizi *et al*., 2019b). All costs were inflated to 2020 values using the consumer price index (Statistics South Africa, 2020a) and converted to 2020 USD (South African Reserve Bank, 2020). Future costs were discounted using an annual 5.0% discount rate per South African guidelines (National Department of Health, 2013).

The costs of the CCT intervention were based on OOP costs associated with seeking diabetes screening and treatment services in the public sector, as the CCT programme was conceptualized to cover these costs. These consisted of direct non-medical (transport) and indirect (loss of wages) costs. In the absence of a diabetes-specific estimate for these costs at the primary healthcare level, an estimate of the OOP costs of seeking care at public health facilities in SA was taken from the literature (Tempia *et al*., 2019). For treatment services, four CCTs were modelled annually (corresponding to the four clinic visits required annually to collect medication and undergo complication screening (Basu *et al*., 2019)), totalling $51 per year. For those attending diagnosis services, a one-off CCT of $13 was modelled.

### Outcomes

#### Cost and health outcomes

Costs and health outcomes were accrued by individuals as they progressed through the model in each CCT programme scenario. Total costs for the population, as well as cost per eligible individual, were calculated over the 45-year time horizon. Costs accrued for each strategy were compared to the costs accrued in the base case (no CCT programme) to obtain the incremental costs for each strategy, and in each eligibility scenario.

Health gains were calculated using DALYs averted due to the CCT programme, using disability weights (Table 1) applied to the length of time spent in each state (Rushby and Hanson, 2001). Incremental cost-effectiveness ratios (ICERs) were computed by dividing the incremental costs by incremental DALYs averted for each strategy, compared to a ‘no programme’ scenario. ICERs were calculated using a health system perspective (OOP costs were not included in this analysis) and were compared to South Africa’s cost-effectiveness threshold of $3015 per DALY averted, to determine cost-effectiveness in the South African setting (Edoka and Stacey, 2020). This supply-side threshold was calculated using the inverse of the marginal product of spending on healthcare in the public sector and provides an estimate of the opportunity cost of healthcare spending in South Africa (Edoka and Stacey, 2020).

Cost-effectiveness of the CCT programme strategies is presented as incremental net monetary benefit (INMB) compared to no programme, using the cost-effectiveness threshold stipulated previously. INMB can be useful to report cost-effectiveness in a study where multiple strategies are being compared; there are non-mutually-exclusive alternatives (such as in a sub-group analysis); and where there is a specified cost-effectiveness threshold (Drummond *et al*., 2015).

#### Financial risk protection

The FRP metric used in this study was the number of cases of CHE averted through the programme. CHE cases were defined as OOP health expenditures exceeding a threshold of the individual’s capacity to pay (Mutyambizi *et al*., 2019b). The capacity to pay for an individual is defined as their total income less subsistence expenditure. In this study, subsistence expenditure was defined as the food poverty line (FPL), or the amount of money required to afford the minimum daily energy intake per month (Supplementary Appendix Table S4) (Statistics South Africa, 2019). Consistent with previous literature, three different threshold scenarios were used in this study to define a CHE case: 10%, 25% and 45% of capacity to pay (Mutyambizi *et al*., 2019b; Feldhaus *et al*., 2021).

All outcomes were disaggregated by income quintile and sex.

#### Sensitivity analyses

Probabilistic sensitivity analysis (PSA) was conducted to assess the impact of uncertainty in the model parameters and the population sample characteristics on the findings (Feldhaus *et al*., 2021). Probability distributions were defined for cost, effectiveness and transition probability parameters, and *n* = 100 sets of parameters were drawn from these distributions to test the effect of uncertainty in the model parameters on the study outcomes. (Distributions and parameters can be found in Supplementary Appendix Tables S1–S3).

One thousand distinct populations were generated to test the effect of potential variability in population characteristics on results. R (version 3.6.2) was used to run the model simulations and sensitivity analyses (R. Core Team, 2020).

In addition, one-way sensitivity analyses were conducted to assess the impact of uncertainty in key parameters, holding other parameters constant. Due to the uncertainty surrounding the effect of the CCT programme on uptake of diabetes services, three univariate sensitivity analyses were conducted: varying the effect of the programme on uptake of both diagnosis and treatment services; varying its effect on uptake of diagnosis services only; varying its effect on uptake of treatment services only. The confidence interval (CI) of the treatment effect parameter was used to set the lower and upper bound in the sensitivity analysis (Owusu-Addo *et al*., 2018). Furthermore, due to the uncertainty of the impact of administrative costs on overall cost-effectiveness, a univariate sensitivity analysis was conducted where administrative costs amounting to 50% of intervention costs were included (Caldés and Maluccio, 2005; García and Saavedra, 2017).

#### Model validation

To determine whether the population simulated by the model reflects the South African population, a simple validation test was conducted. This involved estimating the initial prevalence of diabetes in the simulated population, and comparing it with the latest available estimates of diabetes prevalence, using the South African Demographic and Health Survey 2016 data (Grundlingh *et al*., 2022).

## Results

In the first scenario, where only those in income quintile 1 were eligible for the CCT programme, a total of approximately 6 million individuals in the simulated population were eligible. In the second scenario, where both income quintiles 1 and 2 were included, a total of approximately 12 million of the simulated population were eligible to receive CCTs.

### Costs

The total and incremental costs by CCT strategy for each eligibility scenario are presented in Table 2. When people in income quintile 1 were eligible, providing CCT only for diabetes treatment services resulted in the lowest incremental cost: approximately $67 million total or $11 per person eligible. The incremental costs of providing CCT for both diagnosis and treatment services were similar, with a cost of approximately $12 per person eligible. The strategy of providing CCT only for diagnosis services resulted in the greatest incremental costs: $110 million total, and $18 per person eligible. Including people in income quintile 2 in eligibility for the programme resulted in approximately $1 extra per person eligible in incremental cost (Table 2), with the incremental total cost of the programme increasing, as would be expected when including more people.

**Table 2.** Cost, health and financial risk protection outcomes

		Incremental† cost (USD 2020)		Incremental† DALYs averted			INMB by sex			Incremental† CHE cases averted		
Eligibility scenario	Strategy	Total	Per person (95% CI)	Total	Per person (95% CI)	INMB*	Female	Male	Incremental† OOP costs averted**	10% threshold	25% threshold	40% threshold
**Income quintile 1**	Diagnosis only	110 144 690	18.41 (16.58–20.22)	3 253	0.000544 (−0.00500–0.00500)	−16.77	−24.76	−19.79	N/A	N/A	N/A	N/A
	Diagnosis + treatment	69 380 988	11.59 (9.78–13.40)	65 277	0.0109 (0.00501–0.0149)	21.29	40.06	15.92	8 715 456	104 403	78 080	60 445
	Treatment only	67 071 454	11.21 (9.38–13.02)	59 330	0.00991 (0.00501–0.0150)	18.68	34.87	14.26	3 984 548	34 968	27 812	22 241
**Income quintiles 1 and 2**	Diagnosis only	235 578 120	19.68 (17.83–21.55)	7 487	0.000626 (−0.00497–0.00497)	−17.80	−23.66	−23.80	N/A	N/A	N/A	N/A
	Diagnosis + treatment	154 964 495	12.95 (11.09–14.81)	137 698	0.0115 (0.00503–0.0150)	21.74	36.23	21.22	19 005 489	156 682	84 794	60 738
	Treatment only	148 835 236	12.44 (10.58–14.30)	124 300	0.0104 (0.00503–0.0150)	18.88	31.90	17.96	8 475 607	55 213	30 317	22 246

Abbreviations: DALY: Disability-adjusted life year; INMB: Incremental net monetary benefit; OOP: Out-of-pocket; CHE: Catastrophic health expenditures.

† Incremental compared to the ‘no CCT programme’ scenario.

* Using a threshold of $3 015 per DALY averted.

** Includes direct non-medical (transport) and indirect costs (productivity losses).

N/A: unable to estimate.

### Health outcomes

Table 2 presents the incremental DALYs averted, for each scenario and programme strategy. For both eligibility scenarios, the programme strategies that included treatment services resulted in greater amounts of incremental DALYs averted, compared to the strategy that included diagnosis services only, with the strategy that includes both diagnosis and treatment services averting the highest number of DALYs, for both eligibility scenarios.

### Costs and health consequences

**Figure 2.**
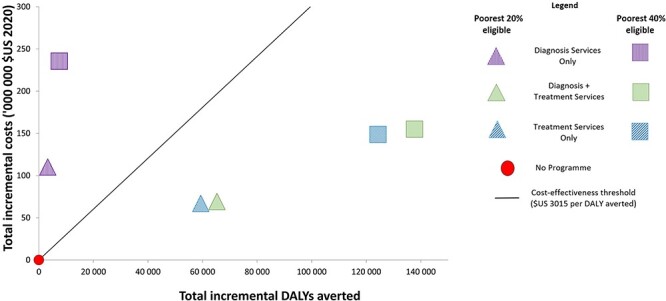
Incremental cost-effectiveness plane showing the incremental costs and incremental DALYs averted, for each strategy and eligibility scenario, compared to no programme

As can be seen in Table 2, offering a CCT programme for both diagnosis and treatment services, for both income quintiles 1 and 2 provides the highest INMB, and thus would be considered the most cost-effective strategy at the South African threshold of $3015 per DALY averted. Relative to men, the INMB for women is higher for each strategy in both eligibility scenarios. The ICERS are plotted on a plane (Figure 2).

### Financial risk protection

FRP benefits were estimated with cases of CHE averted by the CCT programme, compared to ‘no programme’. Incremental CHE cases averted for each catastrophic threshold are presented in Table 2, per strategy and eligibility scenario. Across the three catastrophic thresholds assessed, CHE cases averted are greater when quintiles 1 and 2 are included, compared to only quintile 1. As found with the health benefits, the greatest FRP benefits are found in the CCT strategy where both diagnosis and treatment services are covered, irrespective of CHE threshold or eligibility scenario. The CHE cases averted were greatest among women vs men, for each strategy.

### Sensitivity analysis

**Figure 3.**
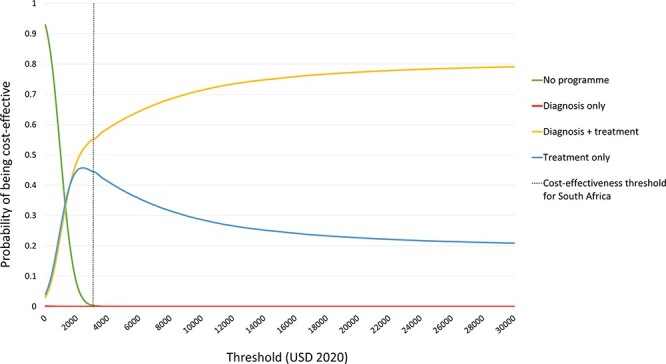
Probabilistic sensitivity analysis and cost-effectiveness acceptability curves

Figure 3 shows the results of the PSA in the form of a cost-effectiveness acceptability curve. Irrespective of the willingness-to-pay threshold, the ‘diagnosis only’ programme strategy is unlikely to be considered cost-effective, relative to the other strategies. At the South African cost-effectiveness threshold, the ‘diagnosis and treatment’ strategy has the highest probability of being cost-effective (approximately 55%), followed by the ‘treatment only’ strategy at approximately 45%. The difference between these two probabilities is only 10%, which does not indicate a high level of superiority, when parameter uncertainty is taken into account. PSA results with ICER planes can be found in Supplementary Appendix Figure S2.

**Figure 4.**
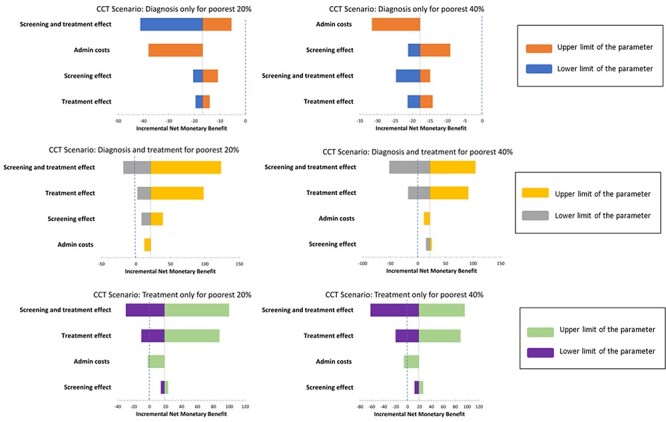
Tornado diagram presenting the one-way sensitivity analysis results for each eligibility scenario (coverage for poorest 20% and poorest 40%) and each CCT programme strategy (‘diagnosis only’, ‘diagnosis and treatment’ and ‘treatment only’)

Note: The tornado diagram ranks the parameters listed on the left in order of their influence on the incremental net monetary benefit, with each parameter varied by the lower and upper range of the parameter.

Figure 4 presents the tornado diagrams resulting from the one-way sensitivity analyses for each CCT programme strategy and eligibility scenario. The INMB is on the *x*-axis with a vertical threshold line at zero showing where the scenario may cross over from being cost-effective to not cost-effective at the South African threshold, as a result of a change in each specified parameter. In the CCT programme strategy where both diagnosis and treatment are covered, costs of administration do not result in a negative INMB, indicating that this strategy would still be cost-effective when these costs are accounted for.

### Model validation

The initial diabetes prevalence in the simulated population was 17.5% and 18.4% for the first and second income quintiles, respectively. While this is slightly lower than the 22% prevalence found in the 2016 South African Demographic Health Survey, diabetes prevalence increases with income in South Africa and it is therefore reasonable to expect that the prevalence in the two bottom quintiles would be lower than the overall prevalence in the population (Mutyambizi *et al*., 2019a; Grundlingh *et al*., 2022).

## Discussion

We assessed the extended cost-effectiveness of three CCT programme strategies to improve utilization of diabetes services in South Africa using a microsimulation Markov model. Both the ‘treatment only’ and ‘diagnosis and treatment’ indicated cost-effectiveness at the South African threshold of $3015 per DALY averted. The highest INMB was found when the CCT programme covered both diagnosis and treatment services, for both the first and second income quintiles. When only treatment services are covered, those with diabetes and undiagnosed continue to access diagnosis services at the existing rate in the population. Consequently, in this scenario, only individuals who have already been diagnosed receive the benefit in medication utilization rate from the CCT programme, thereby reducing the overall cost and health consequences associated with averted complications. The greatest benefits of the programme are seen when the probability of both diagnosis and adherence to treatment are improved using an incentivization strategy. While the costs of this programme strategy are inevitably higher, these are offset by the resultant reduction in direct medical costs associated with diabetes-related complications (Supplementary Appendix Figure S1). Furthermore, this reduction in complications and diabetes-related mortality results in greater incremental health outcomes—represented in this study by the averted DALYs found.

A negative INMB was found when only diagnosis services were covered by the programme, indicating that this programme strategy would not be considered cost-effective at the South African threshold of $3015 per DALY averted. Results of the PSA showed that, relative to the other programme strategies, covering diagnosis services alone would not be cost-effective, irrespective of the threshold adopted (Figure 3). These results are likely due to the consideration that, while an increase in uptake of diagnosis services does increase an individual’s probability of starting diabetes therapy, if it is not followed up with an increase in adherence to diabetes treatment services, then health benefits are not realized to the same extent, as glycaemic control is achieved through adherence to treatment, not merely diagnosis. Fewer complications are averted, relative to CCT programme strategies that include treatment services, leading to greater total incremental costs (Supplementary Appendix Figure S1) and fewer DALYs averted (Table 2).

Findings also showed greater INMB and FRP benefits for women. This is likely to be a function of higher incidence and prevalence of diabetes in women (Sifunda *et al*., 2023). Given the gender inequality that persists in South Africa, particularly with women underrepresented in the labour market, carrying a disproportionately large load in caregiver responsibilities, and with high rates of female-headed households, the relative benefits of the CCT programme could be considered to be an important consideration for equity (Casale and Posel, 2021). In particular, the FRP benefits in the form of CHE cases averted (and the relative benefit for women vs men) demonstrate the potential of the CCT programme to provide benefits to economically vulnerable people in South Africa. The ‘Diagnosis and Treatment’ strategy provides the greatest such benefit, and this equity consideration furthers the argument for consideration of this strategy over the comparators.

Study findings align with the results of a microsimulation model that estimated the impact and cost-effectiveness of scaling up treatment for hypertension, dyslipidaemia and type-2 diabetes in South Africa (Basu *et al*., 2019). While it is difficult to compare ICERs between their study and our own, as a significant proportion of the cost and health benefits found in their study are attributable to hypertension and dyslipidaemia outcomes, Basu and colleagues also found that scaling up treatment for diabetes (and the resultant glycaemic control) averted direct medical costs associated with diabetes-related complications and resulted in averted DALYs (Basu *et al*., 2019). Another study investigating the effect of improved diagnosis, treatment and control of diabetes and hypertension also supports our findings that reductions in complications somewhat offset the increased costs related to higher rates of diagnosis and treatment services (Basu *et al*., 2021). Their finding that blood pressure control was an important strategy to reduce diabetes-related DALYs suggests that combining a CCT programme for hypertension and diabetes services should be considered in future work. This may result in cross-over benefits and result in greater cost-effectiveness.

In an ECEA of using Health Equity Funds to reduce the health and financial burden of diabetes in Cambodia, Feldhaus and colleagues also had similar findings. The scenario where only diagnosis services were covered by the fund showed the least cost-effectiveness, with the scenario covering treatment services showing evidence of cost savings due to complications averted (Feldhaus *et al*., 2021). Some differences in findings occur however, such as differences in healthcare financing mechanisms between South Africa and Cambodia, as well as differences in the structure of the intervention modelled. The Health Equity Fund was aimed at averting OOP direct medical costs faced by patients accessing healthcare in Cambodia, whereas in South Africa, direct medical costs are not borne by the patient, and thus the intervention was targeted at averting direct non-medical and indirect costs faced by patients.

### Limitations

Several limitations apply to our study. First, cost inputs were derived from the literature, standard treatment guidelines and expert clinical advice, as there is no routine collection of cost data in the public health system. In the event of the implementation of National Health Insurance (NHI), data pertaining to costs may be part of monitoring infrastructure, which could provide more robust estimates for models such as this one.

Second, there is a paucity of surveillance data for diabetes in South Africa, particularly when stratifying by income quintile and sex. An update to the 2012 SANHANES-1 study would improve the ability of this model to accurately predict the transitions of individuals through the different health states. Alternative sources for diabetes prevalence and care cascade estimates for South Africa are available, for instance, from the International Diabetes Federation’s Diabetes Atlas; however, these estimates are extrapolated from other countries and thus do not directly use South African data (International Diabetes Federation, 2021).

Third, the lack of available data on the administration costs involved in implementing such a programme means that the costs of the programme are likely underestimated in this study. However, as infrastructure exists within the health system and within the SASSA grants system to conduct means-testing to determine eligibility for grants and for hospital cost exemptions, there are likely efficiencies in using these existing structures to administer the programme. The one-way sensitivity analysis conducted for this parameter demonstrates the potential impact of including administrative costs. The 50% estimate used in the sensitivity analysis is higher than other estimates found in the literature of between 6 and 12% (Grosh *et al*., 2008); however, in the case of the one-way sensitivity analysis, the most conservative estimate was adopted to understand the highest potential impact of administrative costs on cost-effectiveness results (Caldés and Maluccio, 2005). In the CCT strategy where both diagnosis and treatment services are covered, INMB remains above zero when administrative costs are included, indicating that this CCT strategy is cost-effective even if administrative costs are substantial (Figure 4).

Fourth, while the effectiveness input was based on systematic review evidence from Africa, this may not reflect South Africans’ response to such a programme, and thus a South African-specific study would reduce this uncertainty in our results—particularly to determine whether there is differential effect for men compared to women, or for individuals in different income quintiles. This systematic review evidence did not include studies of CCT programmes for increasing utilization of services for non-communicable diseases, which may limit the generalizability of the treatment effect estimate to this study. Additionally, due to lack of evidence, we were unable to differentiate effect size of the CCT on change in utilization rates for diagnosis and treatment services. The impact of the intervention on these rates may differ by service type, but without data on whether the impact is likely to be higher or lower for diagnosis services compared to treatment services, we were unable to adjust the treatment effect. These limitations in the estimation of treatment effect are key limitations to this study; however, the lower bound of the intervention effect was a conservative odds ratio of improved utilization. This uncertainty in treatment effect was therefore factored into the PSA. Furthermore, the one-way sensitivity analyses show how a change in the treatment effect on each of the utilization rates would impact cost-effectiveness. This facilitates interpretation of the results in the presence of parameter uncertainty. Further research in these areas could provide more context-specific and precise estimates of the costs and benefits associated with a CCT programme, as well as their distribution.

Fifth, another limitation, as noted by Feldhaus *et al.*, is that the model used does not account for potential changes in incidence and or mortality rates over the 45-year time horizon (Feldhaus *et al*., 2021). Treatments with increased efficacy may be developed for both diabetes and its associated complications, which would alter various transition probabilities between health states in the simulated population. The time horizon of the study should be long enough to include all relevant cost and clinical endpoints (Weinstein *et al*., 2003). While not covering the full lifetime of the population, the 45-year time horizon used captures the overwhelming majority of the population, considering the average life expectancy of 65 years in South Africa, while also capturing the costs and benefits of the working population (World Health Organization, 2019). While the results should be interpreted with some caution given that the full lifetime horizon is not used, existing results showing that CCT programmes covering treatment services avert complications in the long term, and thus a shorter time horizon is likely underestimating cost-effectiveness of these two scenarios, rather than overestimating it.

Sixth, there may also be policy implication limitations to this study. South Africa is facing ever-increasing constraints on public health spending, and there may be opposition to the budget being used for direct cash transfers to individuals. This may limit the political will required to implement such a programme. Therefore, this ECEA is a useful tool to show that both the health benefits and FRP benefits of such an intervention are likely to improve quality of life for the poorest households in South Africa.

### Recommendations

Based on the results of this analysis, a CCT programme to improve uptake of diabetes services should include both diagnosis and treatment services and be provided to individuals in both income quintiles 1 and 2. The cost-effectiveness threshold used to calculate INMB is not an official decision-making threshold used by policymakers in the country. However, this supply-side threshold provides an estimate of the opportunity cost of the disinvestment required to invest in a new intervention, given a fixed health budget such as the one in South Africa (Vallejo-Torres *et al*., 2016). Opportunity costs are a critical consideration in understanding appropriate expenditure in a health system, and therefore the $3015 per DALY averted threshold, estimated by Edoka and Stacey, is an important indicator of whether an intervention could be considered cost-effective in the South African health system (Sculpher *et al*., 2017; Edoka and Stacey, 2020).

## Conclusion

A CCT programme that covers diagnosis and treatment services was found to be cost-effective in South Africa when provided to the poorest 40% of the population. The ECEA proved useful in determining the distributional benefits of the programme, as well as the FRP benefits. The CCT programme was more cost-effective in women compared to men and provides more FRP benefits to women, compared to men.

## Supplementary Material

czae001_Supp

## Data Availability

The data (microsimulations) that support the findings of this study are hosted on the Wits Core cluster of computers and are available from the corresponding author, (HF), upon reasonable request.

## Reflexivity statement

The majority (5 out of 7) of co-authors are of South African nationality or were based in South Africa during conceptualisation and the greater part of the analysis phase of the research (the first author moved to the UK to pursue further studies during the write-up phase). The work was supported and commissioned by the South African Medical Research Council in an effort to incorporate equity considerations in economic evaluations and thus this work seeks to address questions seen as important to the local South African context. The first author is an ‘Early Career Researcher’ and the mentoring and support provided by the other authors involved in the paper has been foundational to her ambition to become a health economist able to conduct research independently. This work therefore reflects all of the authors’ endeavours to improve the distribution of authors in terms of seniority and regional location, while ensuring robust supervision from experts in the field.
