# Supplementary material for: Extended cost-effectiveness analysis of interventions to improve uptake of diabetes services in South Africa
Source: Health Policy Plan. 2024 Jan 22;39(3):253–67. doi: 10.1093/heapol/czae001 (PMC10929771; doi:10.1093/heapol/czae001)
Supplement: czae001_Supp [file czae001_supp.zip › Supplementary appendix_revised_proof.pdf]

Supplementary Appendix

Contents

CHEERS 2022 Checklist 2

Parameter inputs in probabilistic sensitivity analysis 6

Results 12

References 18

Tables

Table S1. Detailed cost inputs with probability distributions 6

Table S2. Diabetes intervention transition probabilities with probability distributions 8

Table S3. Healthcare utilisation and CCT effect parameters along with their probability distributions 10

Table S4. Income Parameters 11

Table S5. Detailed cost results 13

Table S6. Detailed Health Outcomes Results 15

Table S7. Incremental cost and health outcomes by sex 166

Figures

Figure S1. Incremental cost per person eligible for CCT, by cost category and eligibility scenario 13

Figure S2. Results of probabilistic sensitivity analysis on ICER planes 16

CHEERS 2022 Checklist

Topic	No.	Item	Location where item is reported
**Title**			
	1	Identify the study as an economic evaluation and specify the interventions being compared.	Title, final paragraph of the introduction
**Abstract**			
	2	Provide a structured summary that highlights context, key methods, results, and alternative analyses.	Single paragraph provided after the title page, as instructed by the journal.
**Introduction**			
**Background and objectives**	3	Give the context for the study, the study question, and its practical relevance for decision making in policy or practice.	Disease burden context provided in first 2 paragraphs of the introduction; economic burden and justification for the intervention provided in paragraphs 3-5 and paragraph 8. Decision-making context in paragraph 6. Research aim and objectives provided in the 7th paragraph of the introduction.
**Methods**			
**Health economic analysis plan**	4	Indicate whether a health economic analysis plan was developed and where available.	Health economic analysis plan was not developed
**Study population**	5	Describe characteristics of the study population (such as age range, demographics, socioeconomic, or clinical characteristics).	All three paragraphs in the 'Model inputs' sub-heading in the Methods
**Setting and location**	6	Provide relevant contextual information that may influence findings.	Local context for structure of intervention: 'Intervention structure' sub-heading in Methods section; and 'Model Structure' heading in the Methods section.
**Comparators**	7	Describe the interventions or strategies being compared and why chosen.	5th paragraph in the Methods section
**Perspective**	8	State the perspective(s) adopted by the study and why chosen.	1st paragraph in the 'Model structure' sub-heading

Topic	No.	Item	Location where item is reported
**Time horizon**	9	State the time horizon for the study and why appropriate.	1st paragraph in the 'Model structure' sub-heading
**Discount rate**	10	Report the discount rate(s) and reason chosen.	Final sentence in the 3rd paragraph of the 'Costs' sub-heading
**Selection of outcomes**	11	Describe what outcomes were used as the measure(s) of benefit(s) and harm(s).	All four paragraphs in the 'Outcomes' sub-heading
**Measurement of outcomes**	12	Describe how outcomes used to capture benefit(s) and harm(s) were measured.	All four paragraphs in the 'Outcomes' sub-heading
**Valuation of outcomes**	13	Describe the population and methods used to measure and value outcomes.	All four paragraphs in the 'Costs' sub-heading; Table 1
**Measurement and valuation of resources and costs**	14	Describe how costs were valued.	All four paragraphs in the 'Costs' sub-heading; Table 1
**Currency, price date, and conversion**	15	Report the dates of the estimated resource quantities and unit costs, plus the currency and year of conversion.	2nd last sentence in the third paragraph of the 'Costs' sub-heading
**Rationale and description of model**	16	If modelling is used, describe in detail and why used. Report if the model is publicly available and where it can be accessed.	First paragraph in the methods section; 'Model Structure' sub-heading; Figure 1.
**Analytics and assumptions**	17	Describe any methods for analysing or statistically transforming data, any extrapolation methods, and approaches for validating any model used.	'Model validation' sub-heading for validation approach. The model used a simulated cohort, thus no statistical transformation was required.
**Characterising heterogeneity**	18	Describe any methods used for estimating how the results of the study vary for subgroups.	Final statement in 'Outcomes' sub-heading
**Characterising distributional effects**	19	Describe how impacts are distributed across different individuals or adjustments made to reflect priority populations.	Second and third paragraph of 'Model inputs' sub-heading; Final statement in 'Outcomes' sub-heading

Topic	No.	Item	Location where item is reported
**Characterising uncertainty**	20	Describe methods to characterise any sources of uncertainty in the analysis.	'Sensitivity analyses' sub-heading
**Approach to engagement with patients and others affected by the study**	21	Describe any approaches to engage patients or service recipients, the general public, communities, or stakeholders (such as clinicians or payers) in the design of the study.	Second paragraph of 'Model structure' sub-heading; Second paragraph of 'Costs' sub-heading
**Results**			
**Study parameters**	22	Report all analytic inputs (such as values, ranges, references) including uncertainty or distributional assumptions.	Table 1 in the main text, Tables S1-S4 in the supplementary appendix
**Summary of main results**	23	Report the mean values for the main categories of costs and outcomes of interest and summarise them in the most appropriate overall measure.	Table 2; Costs and health consequences sub-heading.
**Effect of uncertainty**	24	Describe how uncertainty about analytic judgments, inputs, or projections affect findings. Report the effect of choice of discount rate and time horizon, if applicable.	'Sensitivity analysis' sub-heading in Results; Figures 3 and 4 in main text, Figure S2 in supplementary appendix.
**Effect of engagement with patients and others affected by the study**	25	Report on any difference patient/service recipient, general public, community, or stakeholder involvement made to the approach or findings of the study	None.
**Discussion**			
**Study findings, limitations, generalisability, and current knowledge**	26	Report key findings, limitations, ethical or equity considerations not captured, and how these could affect patients, policy, or practice.	Key findings: first 4 paragraphs; Limitations: 'Limitations' sub-heading; ethical/equity considerations: 3rd paragraph; policy recommendations: 'Recommendations' sub-heading.
**Other relevant information**			

Topic	No.	Item	Location where item is reported
**Source of funding**	27	Describe how the study was funded and any role of the funder in the identification, design, conduct, and reporting of the analysis	Title page
**Conflicts of interest**	28	Report authors conflicts of interest according to journal or International Committee of Medical Journal Editors requirements.	Title page

From: Husereau D, Drummond M, Augustovski F, et al. Consolidated Health Economic Evaluation Reporting Standards 2022 (CHEERS 2022) Explanation and Elaboration: A Report of the ISPOR CHEERS II Good Practices Task Force. Value Health 2022;25.
doi:10.1016/j.jval.2021.10.008

Parameter inputs in probabilistic sensitivity analysis

In a similar fashion to the Markov model developed for Cambodia, uncertainty in the parameters and the population sample characteristics was tested to assess the robustness of findings (Feldhaus, Nagpal and Verguet, 2021). Probability distributions were defined for both cost and effectiveness parameters. Tables S1, S2 and S3 present detailed parameter inputs along with the probability distributions used in the probabilistic sensitivity analysis.

Table S1. Detailed cost inputs with probability distributions

Parameter	Description	Base Case Value	Low	High	Distribution (parameters)*	Source
**Public healthcare costs**						
Screening	Unit cost of FPG test	USD 3	1	4	lognormal (0.941, 0.354)	(Diabetes South Africa, 2019; National Department of Health South Africa, 2020)
Laboratory diagnostics	Cost of laboratory services for diagnostic testing	USD 6	3	10	lognormal (1.81, 0.354)	(National Health Laboratory Service, 2018)
Oral anti-diabetic (OAD) therapy	Annual average cost of OAD per patient	USD 23	12	35	lognormal (3.09, 0.354)	(National Department of Health, 2018, 2020)
Insulin	Annual average cost of insulin per patient, including cost of glucometer and metformin	USD 104	52	155	lognormal (4.58, 0.354)	(National Department of Health, 2014, 2018, 2020)
Diabetes management	Annual costs associated with ongoing management of patients with diabetes, including outpatient nurse visit every three months; haemoglobin level check (every three months); lipid labs; annual electrolyte and urea labs; statins, aspirin, ACE inhibitors; retinopathy screening	USD 156	81	244	lognormal (5.02, 0.354)	(National Department of Health, 2018; Basu *et al.*, 2019)
Outpatient visits	Cost of consultation at a primary healthcare facility	USD 13	7	20	lognormal (2.53, 0.354)	(Erzse *et al.*, 2019; National Department of Health South Africa, 2020)

Inpatient visits	Cost of hospitalisation due to diabetes-related complications	USD 1207	604	1811	lognormal (7.03, 0.354)	(Erzse *et al.*, 2019; National Department of Health South Africa, 2020)
Discount rate	Discount rate applied to public spending	0.05				(National Department of Health, 2013)
**Individual expenditures**						
Transport costs						
Outpatient care	Average cost of transport for care seeking at a primary healthcare facility	USD 2	1	3	lognormal (0.470, 0.354)	(Stefano Tempia *et al.*, 2019)
Inpatient care	Average cost of transport for care seeking at a public hospital	USD 12	7	16	lognormal (2.44, 0.286)	(Stefano Tempia *et al.*, 2019)
Indirect costs (productivity losses)	Average loss of wages associated with seeking care in the public sector (adjusted for unemployment rate)					
Outpatient care		USD 11	5	20	lognormal (2.33, 0.394)	(Stefano Tempia *et al.*, 2019)
Inpatient care	These are assigned to the patient costs in the model, but not covered by CCT	USD 36	18	56	lognormal (3.38, 0.300)	(Mutyambizi *et al.*, 2019; Stefano Tempia *et al.*, 2019)
**Intervention cost**						
Conditional cash transfer	Amount given to patients on attending appointment	USD 13				(Stefano Tempia *et al.*, 2019)
Eligible	Income percentile eligible for CCT	0.2/0.4				Assumption
Rand/Dollar Exchange rate	Rand to Dollar exchange rate	ZAR 16				(South African Reserve Bank, 2020)

Table S2. Diabetes intervention transition probabilities with probability distributions

Parameter	Description	Value	Distribution (parameters)	Source
Diabetes diagnosis	Probability of being diagnosed with diabetes	0.4	Beta (9.2, 13.8)	(Stokes *et al.*, 2017)
**Probability of diabetes interventions**				
Oral Antidiabetic Drugs (OAD)	Probability of receiving OAD prescription	0.49	Beta (48.5, 50.5)	(Pinchevsky *et al.*, 2017)
Insulin	Probability of receiving insulin prescription	0.091	Beta (2.92, 29.2)	(Pinchevsky *et al.*, 2017)
Combination	Probability of receiving a combination prescription of OAD and insulin	0.401	Beta (38.1, 57.0)	(Pinchevsky *et al.*, 2017)
OAD to insulin	Probability of transitioning from OAD to insulin therapy	0.04	Beta (0.574, 13.8)	(Ringborg *et al.*, 2010; Feldhaus, Nagpal and Verguet, 2021)
Dietary intervention	Probability of being advised a diet-based intervention	0.018	(1 – probability of being on OAD, insulin or combination)	(Pinchevsky *et al.*, 2017)
Adherence	Probability of adhering to prescribed therapy	0.194	Beta (11.4, 47.4)	(Stokes *et al.*, 2017)
**Effects of therapies on complication rates**				
Diet on all complications	Effect of dietary intervention on diabetes- related complications	0.90	N/A	(Feldhaus et al., 2021, assumption)
Glucose-lowering agents (OAD and insulin):				
Myocardial infarction	Effect of glucose-lowering agents on the rate of myocardial infarction in people with diabetes	0.61	Beta (9.07, 5.80)	(Chaudhury *et al.*, 2017; UKPDS 34, 1998; Feldhaus, Nagpal and Verguet, 2021)
Stroke	Effect of glucose-lowering agents on the rate of stroke in people with diabetes	0.59	Beta (3.76, 2.61)	(UKPDS 33, 1998; Feldhaus, Nagpal and Verguet, 2021)
Nephropathy	Effect of glucose-lowering agents on the rate of nephropathy in people with diabetes	0.3	Beta (3.99, 1.67)	(Chaudhury *et al.*, 2017; UKPDS 33, 1998; UKPDS 34, 1998; Feldhaus, Nagpal and Verguet, 2021)
Retinopathy	Effect of glucose-lowering agents on the rate of retinopathy in people with diabetes	0.68	Beta (19.0, 8.94)	(UKPDS 33, 1998; Feldhaus, Nagpal and Verguet, 2021)

Neuropathy	Effect of glucose-lowering agents on the rate of neuropathy in people with diabetes	0.94	Beta (2.08, 13.2)	(Juster-Switlyk and Smith, 2016; Feldhaus, Nagpal and Verguet, 2021)
Angina Pectoris	Effect of glucose-lowering agents on the rate of angina pectoris in people with diabetes	0.68	Beta (19.0, 8.94)	(UKPDS 33, 1998; Feldhaus, Nagpal and Verguet, 2021)
Peripheral vascular disease (PVD)	Effect of glucose-lowering agents on the rate of PVD in people with diabetes	0.74	Beta (3.34, 1.17)	(UKPDS 33, 1998; Feldhaus, Nagpal and Verguet, 2021)
Heart failure	Effect of glucose-lowering agents on the rate of heart failure in people with diabetes	0.68	Beta (19.0, 8.94)	(UKPDS 33, 1998; Feldhaus, Nagpal and Verguet, 2021)
Hyperglycaemia	Effect of glucose-lowering agents on the rate of hyperglycaemia in people with diabetes	0.68	Beta (19.0, 8.94)	(UKPDS 33, 1998; UKPDS 34, 1998; Feldhaus et al., 2021, assumption)
Diabetes-related mortality	Effect of glucose-lowering agents on the rate of diabetes-related mortality	0.58	Beta (6.87, 4.97)	(Institute for Health Metrics and Evaluation, 2017)
All-cause mortality	Effect of glucose-lowering agents on the rate of all-cause mortality in people with diabetes	0.64	Beta (10.1, 5.66)	(Institute for Health Metrics and Evaluation, 2017)

Table S3. Healthcare utilisation and CCT effect parameters along with their probability distributions

Parameter	Description	Value	Distribution	Source
**Healthcare utilisation for diabetes-related complications**				
Outpatient visit	Probability of outpatient care utilisation for diabetes-related complications	0.351	Beta (3.20, 5.92)	(Shisana *et al.*, 2015; Stokes *et al.*, 2017)
Inpatient visit	Probability of utilisation of hospital services for diabetes-related complications	0.351	Beta (3.20, 5.92)	(Shisana *et al.*, 2015; Stokes *et al.*, 2017)
**Conditional Cash Transfer (CCT) Programme effect**				
Diagnosis	Effect of the CCT programme on probability of seeking diagnostics services	1.31	Lognormal (0.270, 0.0812)	(Yotebieng *et al.*, 2016)
Treatment initiation	Effect of the CCT programme on probability of initiating diabetes therapy	1.31	Lognormal (0.270, 0.0812)	(Yotebieng *et al.*, 2016)
Treatment adherence	Effect of the CCT programme on probability of adhering to prescribed diabetes therapy	1.31	Lognormal (0.270, 0.0812)	(Yotebieng *et al.*, 2016)

*Lognormal parameters: mean and standard deviation of the distribution on the lognormal scale; Beta parameters: shape and scale of the distribution.

In terms of the CCT programme effect parameters, it is important to note that separate effects are reported for treatment initiation and adherence as they are impacting different transition probabilities in the model (Pdt for the initiation on treatment, and the probability of remaining in the ‘Diabetes treatment state’ recurrent arrow for the treatment adherence parameter in Figure 1 in the main text). While they are of the same magnitude, they will impact different individuals in the model depending on which state they are in (‘not on treatment’ or diabetes: treatment’). Thus, these probabilities are not cumulative, they cannot concurrently occur to any individual in the model.

Table S4. Income parameters

**Parameter**	**Description**	**Value (USD 2020)**	**Source**
Income status			
Quintile 1	Income band for the poorest 20% of South Africans	<525	(Southern Africa Labour and Development Research Unit, 2016; Saxena *et al.*, 2019)
Quintile 2	Income band for quintile 2	525-1119	(Southern Africa Labour and Development Research Unit, 2016; Saxena *et al.*, 2019)
Quintile 3	Income band for quintile 3	1119-2275	(Southern Africa Labour and Development Research Unit, 2016; Saxena *et al.*, 2019)
Quintile 4	Income band for quintile 4	2275-5788	(Southern Africa Labour and Development Research Unit, 2016; Saxena *et al.*, 2019)
Quintile 5	Income band for the richest 20% of South Africans	>5788	(Southern Africa Labour and Development Research Unit, 2016; Saxena *et al.*, 2019)
Average monthly income	Average monthly income, according to Gamma distribution of income in South Africa	4702	(Southern Africa Labour and Development Research Unit, 2016; Saxena *et al.*, 2019)
Food poverty line	The amount required to afford the minimum required daily energy intake, per person per annum	497	(Statistics South Africa, 2019; Saxena *et al.*, 2019)

Results

Tables S5 and S6 present detailed cost and health outcomes results. Figure S1 shows, in the form of a graph, the incremental costs broken down into broad cost categories: diagnostic costs; cost of medications; and the cost of complications. This figure demonstrates that both programme scenarios that covered treatment services were able to avert higher levels of costs due to diabetes-related complications compared to the scenario that covered diagnostics services only.

Table S7 shows the cost and health outcome results disaggregated by sex, with the incremental net monetary benefit (INMB) calculated using the South African cost-effectiveness threshold of USD 3015 per DALY averted (Edoka and Stacey, 2020).

Table S5. Detailed cost results

Eligibility	CCT Strategy	Number of individuals eligible	Cost of diagnosis	Cost of treatment	Cost of complications	Total costs	Incremental Costs				
							Costs of diagnosis	Costs of treatment	Costs of complications	Total costs	Per person
**Income quintile 1**	No programme	5 984 458	21 325 943	1 008 834 931	2 592 503 362	3 622 664 235	NA	NA	NA	NA	NA
	Diagnostics only	5 984 458	22 532 107	1 123 881 677	2 586 395 141	3 732 808 925	1 206 164	115 046 746	-6 108 221	110 144 690	18.41
	Diagnostics + treatment	5 984 458	22 285 555	1 137 367 525	2 532 392 143	3 692 045 223	959 612	128 532 595	-60 111 218	69 380 988	11.59
	Treatment only	5 984 458	22 440 783	1 121 581 060	2 545 713 846	3 689 735 690	1 114 840	112 746 129	-46 789 515	67 071 454	11.21
**Income quintiles 1 and 2**	No programme	11 968 916	44 184 443	2 113 182 142	5 458 254 811	7 615 621 396	NA	NA	NA	NA	NA
	Diagnostics only	11 968 916	46 514 784	2 359 054 083	5 445 630 649	7 851 199 516	2 330 341	245 871 941	-12 624 162	235 578 120	19.68
	Diagnostics + treatment	11 968 916	46 068 950	2 388 447 523	5 336 069 418	7 770 585 891	1 884 507	275 265 381	-122 185 393	154 964 495	12.95
	Treatment only	11 968 916	46 423 287	2 353 706 772	5 364 326 572	7 764 456 632	2 238 844	240 524 630	-93 928 239	148 835 236	12.44

Abbreviations: CCT: Conditional cash transfer; NA: Not applicable.
All costs are presented in USD 2020.

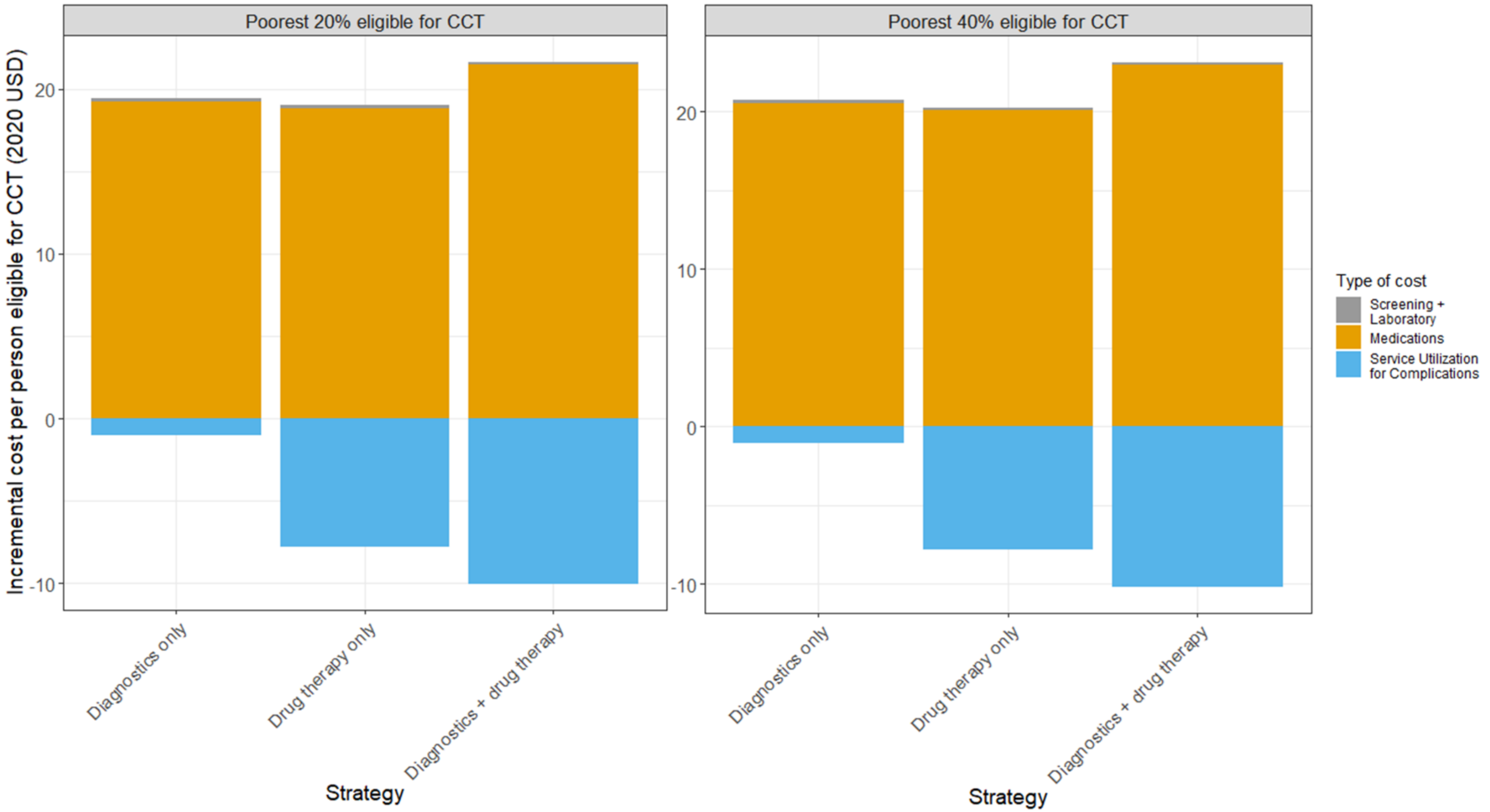

Figure S1. Incremental cost per person eligible for CCT, by cost category and eligibility scenario

Table S6. Detailed Health Outcomes Results

Eligibility	CCT Strategy	Number of individuals eligible	Diabetes-related DALYs	Other DALYs	Total DALYs	Incremental DALYs averted			
						Diabetes-related DALYs	Other DALYs	Total	Per person
**Income quintile 1**	No programme	5 984 458	6 961 252	18 105 446	25 066 698	NA	NA	NA	NA
	Diagnostics only	5 984 458	6 959 991	18 103 454	25 063 445	1 261	1 992	3 253	0.000544
	Diagnostics + treatment	5 984 458	6 913 955	18 087 466	25 001 421	47 297	17 980	65 277	0.0109
	Treatment only	5 984 458	6 917 263	18 090 105	25 007 368	43 989	15 341	59 330	0.00991
**Income quintiles 1 and 2**	No programme	11 968 916	14 073 410	36 217 512	50 290 922	NA	NA	NA	NA
	Diagnostics only	11 968 916	14 070 186	36 213 249	50 283 435	3 224	4 263	7 487	0.000626
	Diagnostics + treatment	11 968 916	13 973 615	36 179 609	50 153 224	99 795	37 903	137 698	0.0115
	Treatment only	11 968 916	13 981 486	36 185 136	50 166 622	91 924	32 376	124 300	0.0104

Abbreviations: CCT: Conditional cash transfer; DALY: Disability-adjusted life year.

Table S7. Incremental cost and health outcomes by sex

Eligibility	CCT Strategy	Women				Men			
		**Incremental total costs**	**Incremental total DALYs averted**	**ICER**	**INMB**	**Incremental total costs**	**Incremental total DALYs averted**	**ICER**	**INMB**
**Income quintile 1**	Diagnostics only	65 392 761	2 635	24 817	-24.76	44 751 929	618	72 433	-19.79
	Diagnostics + treatment	42 633 215	44 963	948	40.06	26 747 773	20 314	1 317	15.92
	Treatment only	40 801 815	40 363	1 011	34.87	26 269 639	18 967	1 385	14.26
**Income quintiles 1 and 2**	Diagnostics only	123 891 755	4 666	26 554	-23.66	111 686 365	2 821	39 587	-23.80
	Diagnostics + treatment	82 907 119	83 280	996	36.23	72 057 375	54 418	1 324	21.22
	Treatment only	78 312 835	75 088	1 043	31.90	70 522 400	49 212	1 433	17.96

Abbreviations: CCT: Conditional cash transfer; DALY: Disability-adjusted life year; ICER: Incremental cost-effectiveness ratio; INMB: Incremental net monetary benefit

Figure S2 shows the output of the probabilistic sensitivity analysis, with the ICERs from each model iteration plotted on an ICER plane. The ellipses within each scatter represent a 95% confidence level for the distribution of the ICERs. As can be seen, the scatter within the ellipses falls wholly above the cost-effectiveness threshold line for the programme scenario where only diagnostic services are covered. Contrary to this, the scatter of ICERs within the ellipses fall wholly below the cost-effectiveness threshold for both programme scenarios where treatment services are covered.

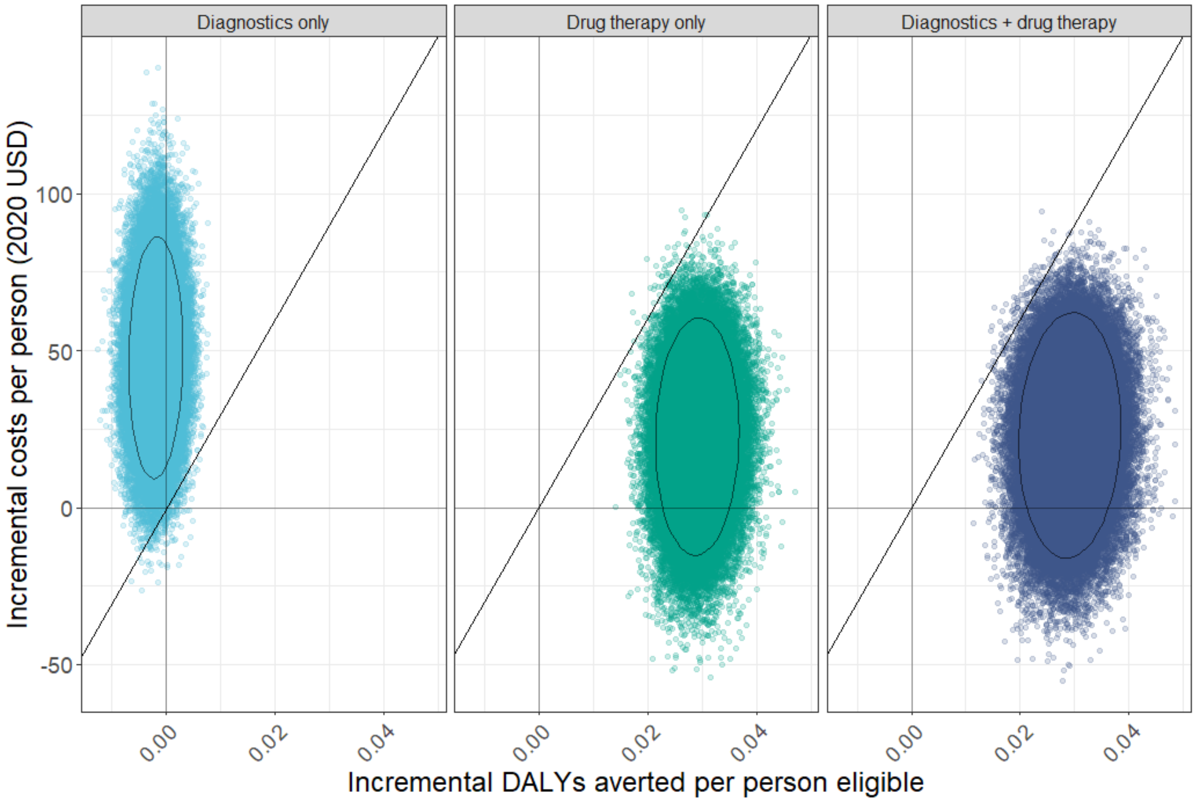

Figure S2. Results of probabilistic sensitivity analysis on ICER planes

References

Basu, S., Wagner, R.G., Sewpaul, R., Reddy, P. and Davies, J. (2019) ‘Implications of scaling up cardiovascular disease treatment in South Africa: a microsimulation and cost-effectiveness analysis’, *The Lancet Global Health*, 7(2), pp. e270–e280. Available at: https://doi.org/10.1016/S2214-109X(18)30450-9.

Chaudhury, A., Duvoor, C., Reddy Dendi, V.S., Kraleti, S., Chada, A., *et al.* (2017) ‘Clinical Review of Antidiabetic Drugs: Implications for Type 2 Diabetes Mellitus Management’, *Frontiers in Endocrinology*, 8, p. 6. Available at: https://doi.org/10.3389/fendo.2017.00006.

Diabetes South Africa (2019) *(Cost) effective blood glucose monitoring*, *DIABETES SOUTH AFRICA*. Available at: https://www.diabetessa.org.za/cost-effective-blood-glucose-monitoring/ (Accessed: 25 November 2020).

Edoka, I.P. and Stacey, N.K. (2020) ‘Estimating a cost-effectiveness threshold for health care decision-making in South Africa’, *Health Policy and Planning*, 35(5), pp. 546–555. Available at: https://doi.org/10.1093/heapol/czz152.

Erzse, A., Stacey, N., Chola, L., Tugendhaft, A., Freeman, M., *et al.* (2019) ‘The direct medical cost of type 2 diabetes mellitus in South Africa: a cost of illness study’, *Global Health Action*, 12(1), p. 1636611. Available at: https://doi.org/10.1080/16549716.2019.1636611.

Feldhaus, I., Nagpal, S. and Verguet, S. (2021) ‘Alleviating the burden of diabetes with Health Equity Funds: Economic evaluation of the health and financial risk protection benefits in Cambodia’, *PLOS ONE*, 16(11), p. e0259628. Available at: https://doi.org/10.1371/journal.pone.0259628.

Institute for Health Metrics and Evaluation (2017) *Global Burden of Disease Study 2017 (GBD 2017) Data Resources - GHDx*. Available at: http://www.healthdata.org/south-africa (Accessed: 2 September 2020).

Juster-Switlyk, K. and Smith, A.G. (2016) ‘Updates in diabetic peripheral neuropathy’, *F1000Research*, 5. Available at: https://doi.org/10.12688/f1000research.7898.1.

Mutyambizi, C., Pavlova, M., Hongoro, C., Booysen, F. and Groot, W. (2019) ‘Incidence, socio-economic inequalities and determinants of catastrophic health expenditure and impoverishment for diabetes care in South Africa: a study at two public hospitals in Tshwane’, *International Journal for Equity in Health*, 18(1), p. 73. Available at: https://doi.org/10.1186/s12939-019-0977-3.

National Department of Health (2013) *Guidelines for Pharmacoeconomic Submissions 2012*.

National Department of Health (2014) *Management of type 2 diabetes in adults at primary care level.* Directorate: Chronic Diseases, Disabilities and Geriatrics. Available at: https://extranet.who.int/ncdccs/Data/ZAF_D1_Management%20of%20type%202%20Diabetes-%20Electronic%20copy%20%202014.pdf.

National Department of Health (2018) *Standard Treatment Guidelines and Essential Medicines List for South Africa: Primary Healthcare Level*. Pretoria South Africa: National Department Of Health.

National Department of Health (2020) 'Master procurement catalogue'. Pretoria, South Africa: National Department of Health. Available at: https://www.health.gov.za/tenders/ (Accessed: 4 February 2021).

National Department of Health South Africa (2020) 'Uniform Patient Fee Schedule 2020'. National Department of Health South Africa. Available at: http://www.health.gov.za/index.php/uniform-patient-fee-schedule/category/652-twenty (Accessed: 20 July 2020).

National Health Laboratory Service (2018) *State price list*. Available at: https://paediatrics.org.za/wp-content/uploads/2023/05/NHLS-State-Price-List-2018.pdf (Accessed: 18 June 2023).

Pinchevsky, Y., Butkow, N., Chirwa, T. and Raal, F. (2017) 'Treatment Gaps Found in the Management of Type 2 Diabetes at a Community Health Centre in Johannesburg, South Africa', *Journal of Diabetes Research*. Edited by F. Barbetti, 2017, p. 9536025. Available at: https://doi.org/10.1155/2017/9536025.

Ringborg, A., Lindgren, P., Yin, D.D., Martinell, M. and Stålhammar, J. (2010) 'Time to insulin treatment and factors associated with insulin prescription in Swedish patients with type 2 diabetes', *Diabetes & Metabolism*, 36(3), pp. 198–203. Available at: https://doi.org/10.1016/j.diabet.2009.11.006.

Saxena, A., Stacey, N., Puech, P.D.R., Mudara, C., Hofman, K., *et al.* (2019) 'The distributional impact of taxing sugar-sweetened beverages: findings from an extended cost-effectiveness analysis in South Africa', *BMJ Global Health*, 4(4), p. e001317. Available at: https://doi.org/10.1136/bmjgh-2018-001317.

Shisana, O., Labadarios, D., Rehle, T., Simbayi, L., Zuma, K., *et al.* (2015) *The South African National Health and Nutrition Examination Survey, 2012: SANHANES-1: the health and nutritional status of the nation*. HSRC Press. Available at: http://repository.hsrc.ac.za/handle/20.500.11910/2864 (Accessed: 21 April 2020).

South African Reserve Bank (2020) *Selected historical rates*. Available at: https://wwwrs.resbank.co.za/webindicators/ExchangeRateDetail.aspx?DataItem=EXCX135D (Accessed: 14 March 2021).

Southern Africa Labour and Development Research Unit (2016) 'National income dynamics study 2014/2015, Wave 4 [dataset].' Pretoria: Department of Planning, Monitoring, and Evaluation, SA Presidency. Available at: https://doi.org/10.25828/f4ws-8a78.

Statistics South Africa (2019) *National Poverty Lines 2019*. P0310.1. Available at: https://www.statssa.gov.za/?page_id=1854&PPN=P0310.1&SCH=7669 (Accessed: 2 August 2021).

Stefano Tempia, Jocelyn Moyes, Adam L. Cohen, Sibongile Walaza, Ijeoma Edoka, *et al.* (2019) ‘Influenza Economic Burden among Potential Target Risk Groups for Immunization in South Africa, 2013-2015’.

Stokes, A., Berry, K.M., Mchiza, Z., Parker, W.-A., Labadarios, D., *et al.* (2017) ‘Prevalence and unmet need for diabetes care across the care continuum in a national sample of South African adults: Evidence from the SANHANES-1, 2011-2012’, *PloS one*, 12(10), pp. e0184264–e0184264. Available at: https://doi.org/10.1371/journal.pone.0184264.

UKPDS 33 (1998) ‘Intensive blood-glucose control with sulphonylureas or insulin compared with conventional treatment and risk of complications in patients with type 2 diabetes (UKPDS 33)’, *The Lancet*, 352(9131), pp. 837–853. Available at: https://doi.org/10.1016/S0140-6736(98)07019-6.

UKPDS 34 (1998) ‘Effect of intensive blood-glucose control with metformin on complications in overweight patients with type 2 diabetes (UKPDS 34)’, *The Lancet*, 352(9131), pp. 854–865. Available at: https://doi.org/10.1016/S0140-6736(98)07037-8.

Yotebieng, M., Thirumurthy, H., Moracco, K.E., Kawende, B., Chalachala, J.L., *et al.* (2016) ‘Conditional cash transfers and uptake of and retention in prevention of mother-to-child HIV transmission care: a randomised controlled trial’, *The Lancet HIV*, 3(2), pp. e85–e93. Available at: https://doi.org/10.1016/S2352-3018(15)00247-7.
